# Multimedia learning with multiple modalities beyond hearing and sight

**DOI:** 10.3389/fpsyg.2026.1433994

**Published:** 2026-09-18

**Authors:** Daniel Laumann, Paul Schlummer, Susanne Heinicke, Stefan Heusler, Stephan Dutke

**Affiliations:** 1 Institute of Physics Education, University of Münster, Münster, Germany; 2 Institute for Psychology in Education, University of Münster, Münster, Germany

**Keywords:** cognitive theory of multimedia learning, integrated model of text and picture comprehension, multimedia learning, multimodal learning, multiple representations

## Abstract

Education and educational research fundamentally rely on advancing learning via multiple representations and comprehensive technological developments that address various sensory modalities. For a more profound understanding of learning processes associated with multimodal learning, psychological theories and models are essential. For the last two decades, most of these processes have been traced to the cognitive theory of multimedia learning and the integrated model of text and picture comprehension, both of which focus on bimodal learning with words and static or animated pictures. However, the field lacks a more comprehensive theoretical approach and more empirical evidence for principles of multimedia learning that address multimedia learning beyond common representations and modalities. Therefore, we propose implications for expanding existing theories by describing learning that involves multiple external representations and addresses various sensory modalities beyond just hearing and sight. In addition, for this theoretical extension, we derive implications for expanded and adapted principles of multimedia learning that include further sensory modalities. Overall, the extension and implications presented here represent drivers for future research on multimodal learning with multiple representations.

## 1 Introduction

Over the past two decades, educational research has increasingly addressed learning with multiple external representations to present objects, such as learning materials and learning environments (defined analogous to, e.g., Rau, 2020). Numerous studies have shown that integrative learning materials that combine different types of external representations open new pathways to support learning. Such representations that can lead to positive learning effects include learning with texts enriched with images (Plass et al., 2003; Schüler et al., 2011b), learning in augmented reality and virtual reality (Milgram et al., 1994) environments (Ibáñez and Delgado-Kloos, 2018; Chang et al., 2022; Xu et al., 2022) or learning with dynamic animations (Höffler and Leutner, 2007; Berney and Bétrancourt, 2016). Also, in science education (for biology, e.g., Won et al., 2014; for chemistry, e.g., Rau, 2015; for physics, e.g., Müller et al., 2017), learning via experiments employs multiple external representations, such as physical activities, the use of gestures and the use of symbols or sounds (Vigliocco et al., 2014).

In each these learning scenarios (and for each of these learning materials), different external representations are used that address different sensory modalities: sight, hearing, smell, taste or the somatosensory system, which includes haptic, tactile, proprioceptive and thermoceptive sensations, according to a typical classification by Møller (2003) and Ten Donkelaar et al. (2020). Therefore, as argued by Barsalou (1999), human cognition and, thus, learning are anchored in a diverse range of sensory systems. So, focusing on these diverse sensory systems represents a way to explain learning beyond traditional textbooks and multimedia.

For many learning media, such as books or “traditional” multimedia applications such as interactive simulations, understanding learning processes requires psychological theories and models. As such, since the early 2000s, the cognitive theory of multimedia learning (CTML; Mayer, 2022) and the integrated model of text and picture comprehension (ITPC; Schnotz and Bannert, 2003) have guided a large body of empirical research on learning with multiple external representations and diverse sensory perceptions and have strongly informed the design of learning materials.

However, empirical investigations derived from these theories have focused mainly on seeing and hearing as sensory modalities, leaving other perceptions largely unexplored. This theoretical focus of CTML and ITPC raises important questions for educational research and practice, since, in many instructional contexts like augmented reality, virtual reality and learning with experiments in science education, learners encounter learning content through additional perceptions, such as tactile interaction with materials or bodily engagement with physical phenomena. Here, it remains unclear whether and how conclusions drawn from existing theories can be transferred to such sensory modalities, as systematic investigations addressing these perceptions are still scarce.

Against this background, the present work examines an extension of theories on learning with multiple external representations that engage learners in a broader range of perceptions. Rather than challenging established theories, this work aims to complement them by providing an extended perspective that accounts for external representations beyond predominantly verbal and visual representations. Such an extension could be of interest for a wide range of didactic and pedagogical perspectives: from learning with real objects to learning with virtual enhancements such as augmented reality or virtual reality, for example, in the field of science education, to disciplines in which movement is a fundamental component of teaching and learning processes, such as in the arts or sports. The value-added research studies in connection with virtual reality called for by Mayer et al. (2023), among others, require expanded theoretical foundations in order to form hypotheses. This paper develops the theoretical considerations in two steps: First, we present an extension of existing theories on learning with multiple external representations, addressing diverse forms of learning with external representations. Second, from this theoretical extension, we derive implications for principles of multimedia learning. Here, we discuss how existing principles of multimedia learning may need to be adapted to account for learning materials that involve a richer set of external representations.

## 2 Theoretical background

The key ideas and principles for describing multimedia learning processes include the concept of (a) multiple coding in memory according to Paivio’s dual coding theory, which discusses verbal stimuli as “logogens” and non-verbal stimuli as “imagens” (Paivio, 1986; Clark and Paivio, 1991); (b) multiple memory systems, as discussed, for example, in theories by Atkinson and Shiffrin (1968) and Baddeley (1986, 2000); (c) multiple types of cognitive load and the limited capacity of the working memory system, as discussed, for example, by cognitive load theory (Chandler and Sweller, 1991; Kirschner et al., 2022; Sweller, 2023); (d) distinctions between codes and modalities (e.g., Scheiter et al., 2018); and (e) the concept that learning is an active, constructive mental process engaged in by the individual (Wittrock, 1974). Against this background, the two most widely acknowledged theories that describe multimedia learning with external representations are the cognitive theory of multimedia learning (CTML; Mayer, 2022) and the integrated model of text and picture comprehension (ITPC; Schnotz and Bannert, 2003).^1^

### 2.1 Cognitive theory of multimedia learning (CTML)

The CTML aims at describing learning processes based on verbal and pictorial information (Mayer, 2022). According to Mayer (2022, p. 1), learning with multiple external representations “occurs when students receive information presented in more than one mode, such as in pictures and words” (see Figure 1). The processing of information is assumed to take place in two separate channels with a sensory system corresponding to the auditory or visual modality. Words are perceived as narration through the ears or as written text through the eyes, while pictures are perceived through the eyes. This information is recoded, separately organized and processed in working memory in line with the dual coding paradigm, subsequently forming a verbal or pictorial mental model (Mayer, 2022). In addition, existing knowledge must be integrated into the development of the mental model and aligned with it. However, with regard to the duality in information processing, further considerations of the CTML have also referred to additional sensory modalities, but these are not elaborated upon beyond merely being mentioned as further elements of sensory memory (auditory and visual, but also tactile, olfactory and gustatory; Moreno and Mayer, 2007).

**FIGURE 1:**
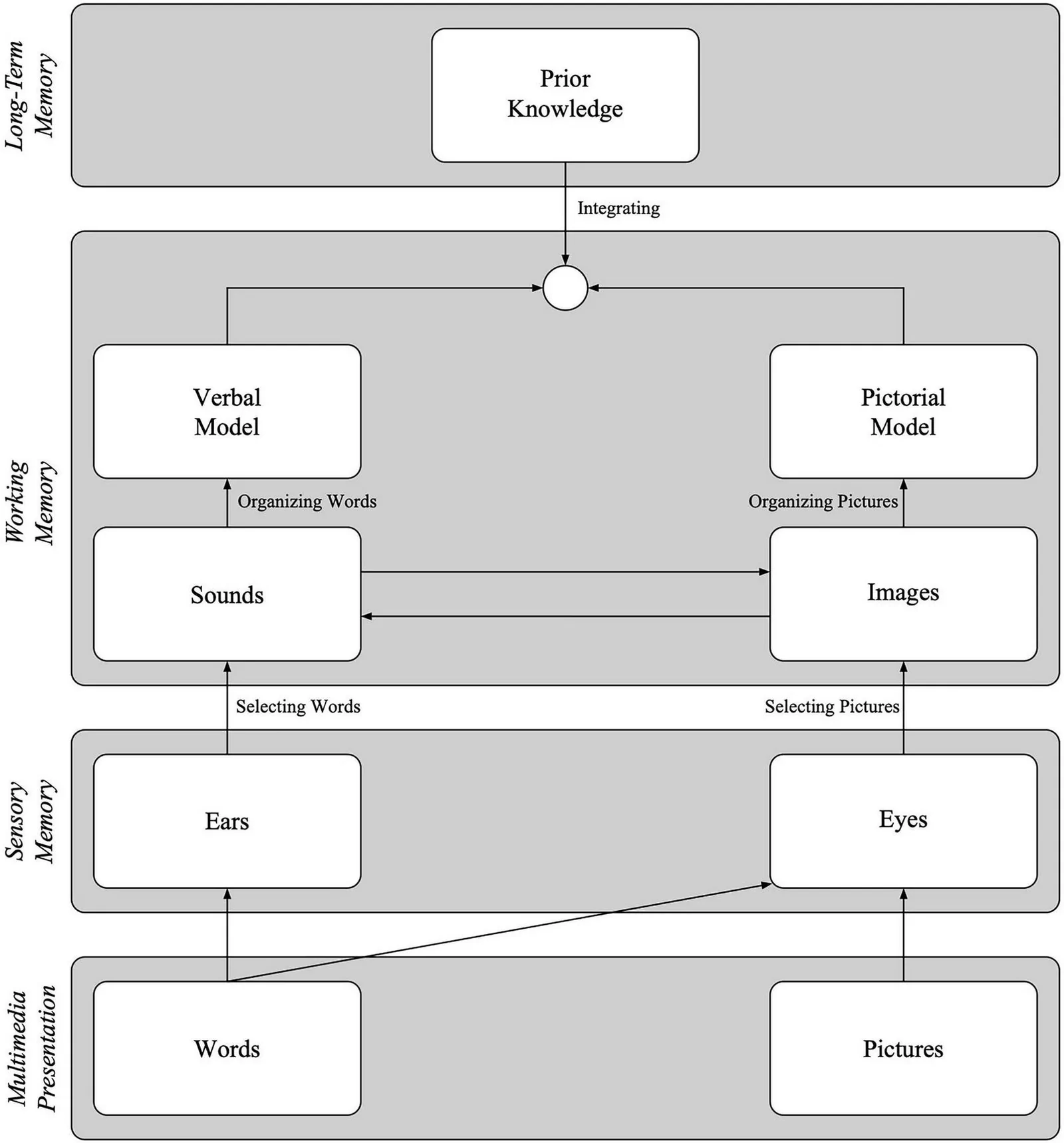
Schematic presentation of the cognitive theory of multimedia learning (CTML; based on Mayer, 2014, p. 52).

The CTML synthesizes and explains various empirical phenomena that have become commonly known as principles of multimedia learning (Mayer, 2022). These principles indicate which arrangements of external representations are expected to foster learning and explain why defectively designed learning environments adversely affect learning processes. For example, the contiguity principle states that verbal and pictorial representations should be presented closely together (spatial contiguity; Ginns, 2006; Holsanova et al., 2009) and simultaneously (temporal contiguity; Baggett and Ehrenfeucht, 1983; Ginns, 2006). The spatial and temporal proximity helps students use their cognitive resources effectively by reducing extraneous cognitive load and ensures that relevant information is presented in working memory at the same time, which facilitates their integration (Mayer, 2022). Empirical studies have substantiated the spatial contiguity principle (e.g., Ginns, 2006; Holsanova et al., 2009) and, to some extent, the temporal contiguity principle (e.g., Baggett and Ehrenfeucht, 1983; Ginns, 2006), but other studies were not able to support a general temporal contiguity principle and referred to the need for more precise definitions of boundary conditions (e.g., Schüler et al., 2012).

Another example of such principles is the modality principle. This principle states that “people learn more deeply from pictures and spoken words than from pictures and printed words” (Mayer, 2009, p. 200) and has been investigated empirically (e.g., Mayer and Moreno, 1998; Moreno and Mayer, 1999). This notion of “the” modality principle has been received and applied countless times and implies a certain kind of universal superiority of multimodal educational materials. Yet, recent findings have indicated “that the ‘modality effect’ is rather an ‘umbrella term’ for seemingly similar observations based on very different processing mechanisms” (Schnotz, 2011, p. 275) and have proven that many boundary conditions exist (e.g., Schüler et al., 2011a; Schmidt-Weigand, 2011). Certain studies have even indicated that specific conditions can actually reverse the modality effect (Lindow et al., 2011; Tabbers and van der Spoel, 2011). Appropriate boundary conditions appear to be fundamentally central to the validity of all principles of multimedia learning (Mayer, 2022).

Another principle that is fundamental to multimedia learning is the multimedia principle itself. This principle states that learning from words and pictures is more effective than learning from text alone (Mayer, 2009) and has been empirically investigated in numerous studies (e.g., Mayer et al., 1996; Höffler and Leutner, 2007). However, other studies provide evidence that learning with texts and images is not always, under all circumstances, more effective than learning with texts alone (Schnotz and Bannert, 2003).

### 2.2 Integrated model of text and picture comprehension (ITPC)

In light of such empirical findings that contradict the CTML and show that images and text are not always more conducive to learning than text alone (Schnotz and Bannert, 2003), it seems necessary to consider the ITPC as another theoretical basis for multimedia learning, as it focuses more strongly on the integration of long-term memory and working memory. Figure 2 shows the ITPC presented by Schnotz (2022).

**FIGURE 2:**
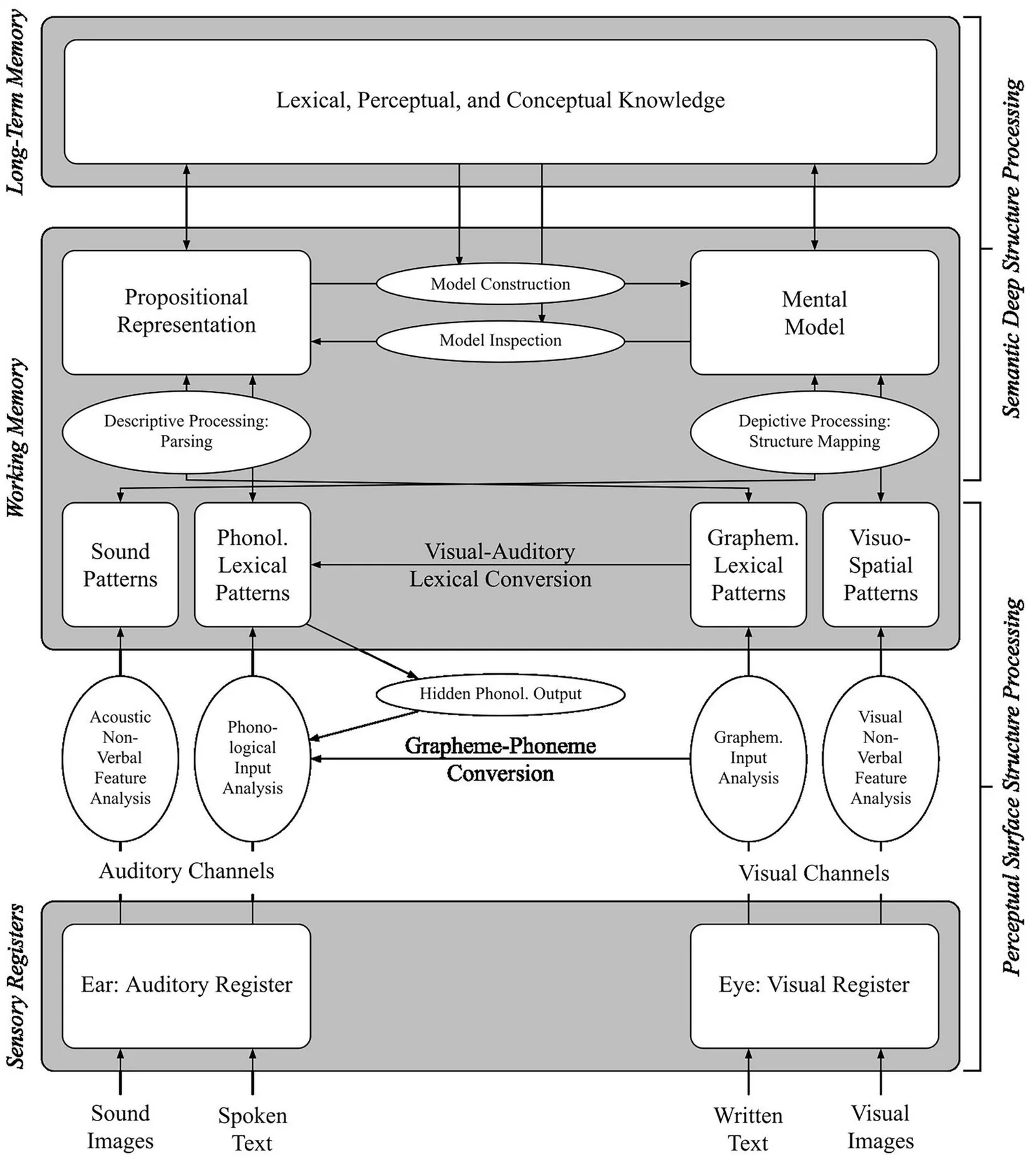
Schematic presentation of the integrated model of text and picture comprehension (ITPC; from Schnotz, 2022, p. 89).

Unlike the CTML, stimuli in the ITPC extend beyond words and pictures to include sounds. In processes that are also fundamentally separate, perceptual surface structure processing then takes place through the analysis of acoustic sound features and phonological inputs or visual pictorial features and graphemic inputs, whereby grapheme-phoneme conversion processes are possible. After the transition to working memory, descriptive processing and forming propositional representations take place in the acoustic/descriptive branch (Figure 2, left side), while a mental model is formed following depictive processing in the visual/depictive branch (Figure 2, right side). These processes take place as part of semantic deep structure processing. Whereas the propositional representation is understood as a logical representation of assertions, the mental model contains more extensive representation of the presented information (Glenberg et al., 1987). Furthermore, the propositional representation supports the construction of the mental model as “a typical instance of what is described” in the given external representation (Schnotz and Bannert, 2003, p. 146).

As a concluding step of semantic deep structure processing, as the learner integrates the propositions, the mental model and prior knowledge, new information may emerge that can be represented propositionally and, thereby, elaborate or revise the propositional basis. In this sense, the mental model is a complex depictive representation that integrates all information and can be mentally manipulated by the learner according their relations; this could be called a “based imagination.” Hence, the mental model is not simply sensory specific as a visual image, and its inspection and mental manipulation can verify propositional representations and lead to new propositions. Contrary to the CTML (Mayer, 2022), the ITPC postulates that comprehension emerges not only from perceptual and semantic processes within different branches but also from interactions between the representations in the two processing branches (Schnotz and Bannert, 2003; Schnotz, 2022). Seufert (2003) stated that learners often struggle to link information within each channel, meaning that they struggle to establish coherence between components of the text or picture (intra-representational coherence) as well as between the verbal and pictorial mental models with each other and with prior knowledge (inter-representational coherence).

In addition, according to Schnotz (2014) and with respect to the ITPC, the cognitive processes used in analyzing acoustic sound features and phonological inputs as well as in analyzing visual pictorial features and graphemic inputs also involve pre-knowledge-guided, top-down processes that are influenced by lexical, perceptual and conceptual knowledge or specific abilities. This claim is backed by studies that have examined the influence of cognitive abilities (Lachmeyer et al., 2007) or representational knowledge (Renkl et al., 2013) on such processes. These types of knowledge also inform and enable the functioning of all lower-order processors, as detailed by Erlebach and Frank (2022).

Just as with the CTML, the ITPC mentions but does not deal further with modalities beyond hearing and seeing (Schnotz and Bannert, 2003).

Our presentation of the CTML and ITPC indicates that both approaches describe underlying processes of learning with texts and pictures in broadly comparable ways. For example, they align with respect to dual coding, and central ideas of the CTML are incorporated into the ITPC (Erlebach and Frank, 2022). However, the two approaches also differ in several aspects. In particular, the ITPC accounts for knowledge-based, top-down processes, unlike CTML, as explained in detail by Erlebach and Frank (2022), and the ITPC emphasizes interactions between the processing branches to be particularly relevant for mental model construction. However, for both theories, aspects of learning with multiple representations involving sensory modalities beyond hearing and vision are only mentioned in passing; they are not discussed in detail. In addition to integrating the central claims of both theories, it therefore seems reasonable to pursue such a theoretical extension.

## 3 Current state and research gap

Looking at the theoretical foundations of multimedia learning (CTML, ITPC), it is clear that these theories consider hearing and seeing as sensory modalities and, consequently, acknowledge a certain spectrum of external representations. While a later version of CTML did consider a wider range of sensory modalities (Moreno and Mayer, 2007) and has also discussed affective and motivational effects in multimedia learning (Astleitner and Wiesner, 2004; Schneider et al., 2022) that have led to advances in the field, for example, with respect to the different effects of decorative and seductive graphics on cognitive and affective-motivational variables (e.g., Rey, 2012), the CTML still focuses on symbolic and iconic external representations; it has focused less on how additional external representations might affect the CTML’s structure. The same applies to the ITPC, which also refers to other modalities without explicitly taking them into account (Schnotz and Bannert, 2003).

However, while the CTML and ITPC have greatly informed the design of materials catering to auditory and visual learning, an extension of existing theories toward a broader spectrum of external representations seems necessary for many areas, for example, learning with real objects, learning with virtual enhancements, such as augmented reality or virtual reality, as well as learning with and from movements, which represent fundamental components of teaching and learning processes in many disciplines such as the arts or sports.

This article discusses a theoretical extension of theories on learning with multiple external representations that address various sensory modalities.^2^ This extension does not aim to disprove existing theories but rather offers a complementary focus, acknowledging the diversity of external representations involved in learning. Thus, this contribution attempts to address the following questions:

1. How could existing theories on learning with multimedia be extended?
2. What implications for principles of multimedia learning can be derived when one considers multiple external representations and multiple sensory modalities (hearing, sight, smell, taste, touch, somatosensory)?

## 4 Multimedia learning with multiple modalities beyond hearing and sight

In the following sections, we derive an extension of theories on multimedia learning (see section 4.1) and implications for specific principles of multimedia learning (see section 4.2). In describing the extension of the theories (section 4.1), we seek to formulate theoretical statements regarding learning with multiple modalities beyond hearing and sight—going beyond CTML and ITPC—based on existing theoretical and empirical findings, while ensuring that these statements remain consistent with the existing theoretical framework. In the implications for specific principles of multimedia learning (section 4.2), we then consider the extent to which these theoretical ideas, when applied to existing principles of multimedia learning, provide insights that may form the basis for future research questions and studies to advance the field provide insights into which research questions could be addressed in future research.

Before describing the theoretical principles of multimedia learning, it is important to clarify some basic terms: Multimedia learning provides various *external representations* that represent external objects such as learning materials and environments (defined analogous to, for example, Rau, 2020). As explained by Ainsworth (2014), external representations provide different types of *stimuli*, for example, visual, auditory or tactile stimuli, that are perceived and selected through different *sensory modalities* (hearing, sight, smell, taste, touch, somatosensory system) in modality-specific sensory memory stores (iconic memory, echoic memory, haptic memory) and can contribute to the construction of *internal representations*. Here, sensory modalities refer to distinct sensory channels involved in perceiving stimuli. Furthermore, internal representations, again with respect to Rau (2020), represent internal or external objects. In the following, the internal representations belonging to the verbal channel of CTML and the descriptive branch of ITPC are referred to as *descriptive representations*. In line with Schnotz’s (1994) reasoning, descriptive representations consist of conventionally chosen signs that describe their represented, whereas depictive representations have some structural similarity with what is represented. The representations belonging to the pictorial channel of CTML and the depictive branch of ITPC are referred to below as *depictive representations* and represent a form of analogous representations. We also include among these, with reference to Barsalou (1999), embodied representations as another type of internal representation, as explained in the following in greater detail.

### 4.1 Extension of current theories on multimedia learning

To extend the theories on multimedia learning, we followed the course of information processing according to the structure shown in Figure 3. For our extension, and consistent with CTML and ITPC, in the first step external representations provide stimuli that are perceived by sensory modalities (4.1.1.), followed by an initial processing phase in working memory (4.1.2.) before various internal representations are formed and organized in the form of mental models (4.1.3.). Figure 3 provides an overview of such a theoretical extension. Sections 4.1.1 through 4.1.3 discuss empirical findings that contribute to expanding the theoretical foundation of learning with multiple representations. Based on these empirical findings, ideas are developed regarding how they might be incorporated into an extended theory. In addition, Section 4.1.4 provides a summary of how this applies to a specific learning scenario—in this case, from the field of natural sciences—which exemplifies and consolidates the statements of the theoretical extension.

**FIGURE 3:**
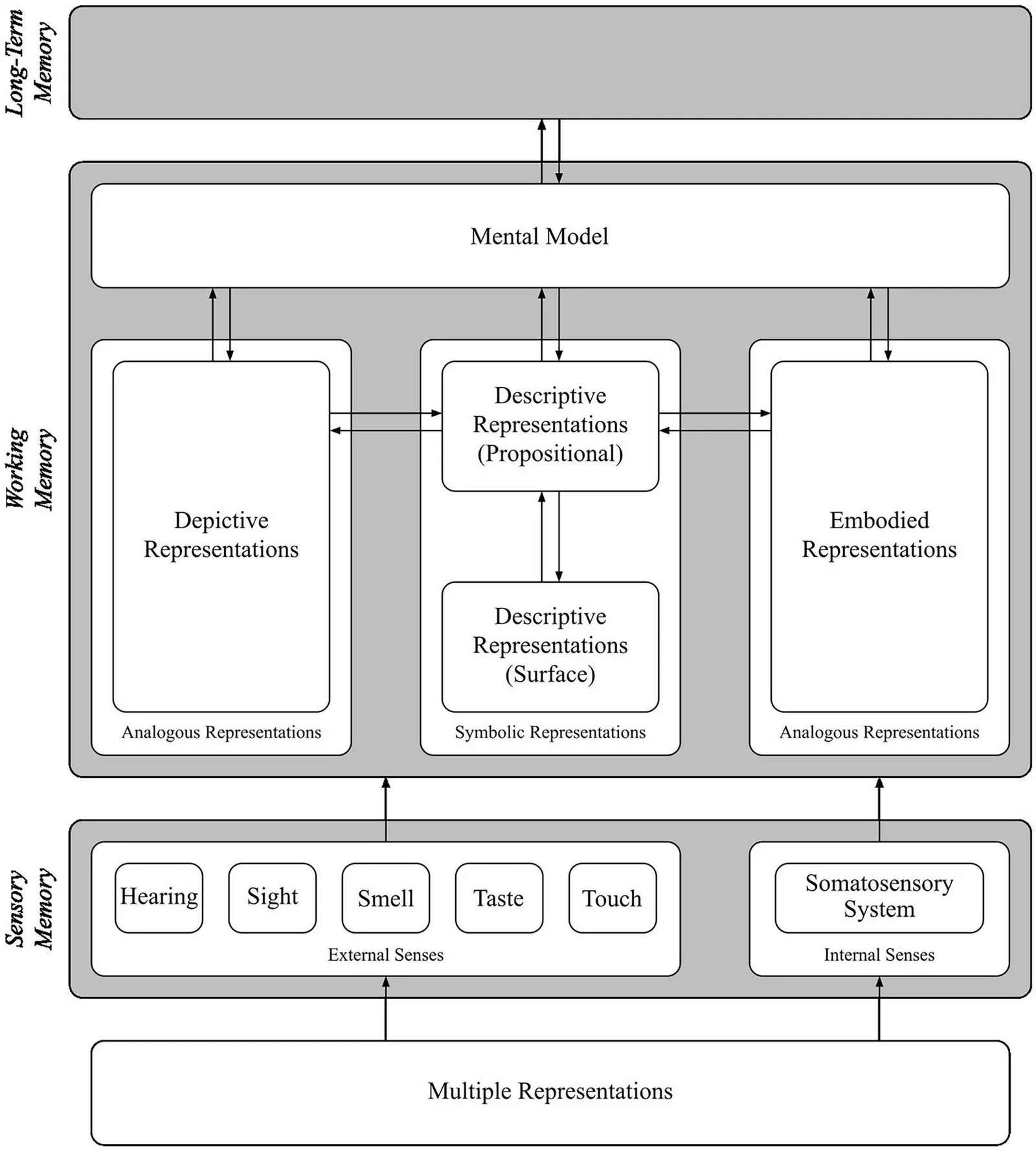
Schematic presentation of a potential adaptation and extension of existing theories on multimodal learning as a theoretical framework.

The complexity of Figure 3 and the theoretical extension discussed below deliberately do not correspond to the high complexity of the current ITPC, as numerous open questions exist and must first be answered based on an initial overarching theoretical extension before one can further differentiate the theoretical approach presented here.

#### 4.1.1 Perceiving stimuli with external and internal senses involving modality-specific sensory memory stores

Stimuli from multiple representations are perceived by sensory modalities; for external stimuli these sensory modalities comprise five senses (hearing, sight, smell, taste, touch), while sensing internal stimuli involves the somatosensory system (haptic, tactile, proprioceptive and thermoceptive sensations). The sensory memory retains information for short periods before some of this information is picked up for further processing in working memory (Coltheart, 1980; Cowan, 2015). Just as the range of sensory modalities extends beyond hearing and seeing to include other senses, sensory memory is also understood to consist of distinct modality-specific systems (Lawson et al., 2016). Of interest are three components of sensory memory: iconic memory, which refers to storing visual information (Sperling, 1960; Walsh and Thompson, 1978; Öğmen and Herzog, 2016; Finley, 2025), echoic memory, which describes storing auditory information (Crowder, 1976; Darwin et al., 1972; Watkins and Watkins, 1980; Kinukawa et al., 2019), and haptic memory, which involves temporarily storing tactile information (Lederman and Klatzky, 2009). Although this three-part division is often explicitly mentioned, it is assumed that there are additional specific systems for other sensory modalities (Finley, 2025). Initial investigations have indicated, for example, that sensory memory can store gustatory (Laureati et al., 2011) and odor-related information (Galán et al., 2006).

Particularly relevant for extending current theoretical foundations are haptic memory and those components of sensory memory that go beyond iconic and echoic memory, as these have not been explicitly addressed in the CTML or the ITPC. Regarding haptic memory, the present literature does refer to haptic memory as a part of the sensory memory and sometimes discusses a type of haptic short-term memory or working memory (Miles and Borthwick, 1996). Yet, distinguishing different types of haptic memory is difficult, as indicated by studies measuring the duration of tactile stimuli storage. For example, an early study by Melzack and Eisenberg (1968, p. 445) discussed “skin sensory afterglows” lasting for minutes after the initial stimulation. Additional investigations measured the preservation of tactile stimuli for different periods of time ranging from 1 s up to more than 1 min (Gilson and Baddeley, 1969; Shih et al., 2009). Based on a comprehensive review of the literature on haptic memory phenomena, Gallace and Spence (2009) proposed a number of functionally distinct but related sub-systems at all memory stages, namely sensory memory, working memory, and long-term memory. Thus, empirical findings suggest that haptic memory exists, meaning it should be considered when extending the theoretical framework for learning with multiple representations.

Furthermore, studies have also indicated that stimuli addressing the somatosensory system are processed in working memory (e.g., Jones and Henriques, 2010; Karnath et al., 2002; Yamamoto and Shelton, 2005) and, therefore, contribute to internal models of “somatosensory joint space” (Cuppone et al., 2018, p. 184) as well as to information stored in long-term memory (e.g., Howard et al., 2013). Along these lines, Schnotz and Bannert (2003) argued that mental models can be constructed not only based on seeing, hearing, and touch but also based on proprioceptive perceptions, and researchers have argued that such further modalities should be considered in connection with the CTML (Moreno and Mayer, 2007). In both cases, however, these additional sensory modalities are mentioned but not thoroughly discussed. Overall, these arguments suggest that the complete spectrum of perceptions arising from sensory modalities and emerging in sensory memory should be accounted for through individual stores to describe them appropriately.

#### 4.1.2 Surface processing of internal representations in working memory

In accordance with the CTML and ITPC and in line with dual coding theory, various internal representations can be distinguished as part of cognition in working memory. “Traditionally conceived, [these] representations are context invariant, disembodied, and static” (Prinz and Barsalou, 2000, p. 52). In addition, the literature contains references to other representations, namely embodied representations that are grounded in modal systems for perception, action, and introspection (Barsalou, 2008). Empirical evidence also suggests that certain concepts are reflected in cognitive process in the form of corresponding embodied representations (e.g., Fu and He, 2025; Wilkowski et al., 2009). Similarly, literature on this topic also discusses which approaches seem valid for characterizing internal representations in cognitive processes and whether internal representations are fundamentally suitable for describing cognitive processes (e.g., Palmiero et al., 2019; Prinz and Barsalou, 2000).

In including embodied representations, this extension of the CTML and ITPC as a theoretical framework for describing learning with multiple representations is based on an integrative approach, which is also reflected in the literature: “An integrative view of the format of mental imagery, one that combines a representation-based approach with an action-based approach, seems possible and increasingly required” (Palmiero et al., 2019, p. 282). Thus, the proposed extension of the theories draws on the depictive (analogous) and descriptive (symbolic) representations introduced by the ITPC and adds embodied representations as another form of analogous representations. To preserve information, embodied representations do not employ arbitrary signs (as descriptive representations) or signs that are structurally like their referents (as depictive representations); instead, they involve a related bodily state and its sensation (Wilson, 2001). For example, perceiving the weight of an object may be represented in the bodily feelings that occur when one attempts to lift the object with one’s hands. In this way, stimuli addressing the somatosensory system may also contribute to working memory and long-term memory representations of the learning content.

The idea of adding embodied representations seems to be compatible with Baddeley’s (1986, 2007) multicomponent model of working memory, which assumes three types of representations: (a) the visuo-spatial sketchpad is assumed to register pictorial information, which may be part of internal depictive representations such as visual images (Baddeley, 2003; Della Sala et al., 1999), (b) the phonological loop is assumed to register speech-like memory traces that are part of an internal descriptive representation, such as words or sentences that are rehearsed verbally or subvocally (Baddeley, 2003, 2010), and (c) the episodic buffer “in which the various components of working memory, each based on a different coding system, can interact through participation in a multidimensional code, and can interface with information from perception and long-term memory” (Baddeley, 2010, p. R138). Furthermore, and also in line with Baddeley (2003), translations between different types of representations are potentially possible; this does not mean that they are possible in all cases and that they require equal amounts of resources. Based on findings related to CTML and ITPC, the complexity and strain of (cognitive) translational processes may depend on (a) the complementarity of information as well as (b) the individual learner’s prior knowledge.

Recently, studies have begun to explore the effects of embodied representations on mental model construction (Ginns et al., 2016). In a more general perspective, Engelkamp and Zimmer (1994, see also Engelkamp et al., 2004, 2005) provided ample evidence that embodied representations play an important role in organizing long-term memory and recollecting verbally described vs. self-performed actions. There are also studies showing that emotions, such as anger, can be represented in an embodied manner within cognitive representations (Wilkowski et al., 2009). A meta-analysis by Fu and He (2025) also discussed evidence for the importance of embodied representations in numerical cognition. Along these lines, embodied representations are considered particularly important for science education and contemporary technologies relevant for teaching and learning (e.g., Lindgren and Johnson-Glenberg, 2013). Thus, embodied representations may potentially supplement descriptive and depictive representations in forming mental models. Nonetheless, the effects of interactions among descriptive, depictive, and embodied representations in learning processes remain to be explored.

#### 4.1.3 Organizing information from internal representations in mental models

In accordance with ITPC (Schnotz, 2022), it can be hypothesized that descriptive and analogous (depictive and embodied) representations are processed in three different pathways.

Descriptive representations require sub-semantic processing, where the analysis of the symbol structures results in an internal representation of the surface structure, for example, of a text. Immediately after an initial basis has been created through sub-semantic processing, the semantic processing starts, which then proceeds in parallel and interacts (Shallice, 1993; Kouider and Dehaene, 2007). The surface representation formed initially is the basis for a semantic analysis, and this results in a net of propositions (propositional representation), which represents the meaning of the external representation (Graesser et al., 1997; Johnson-Laird, 1983; Kintsch and van Dijk, 1978). By contrast, such two-stage processing is not required for external iconic representations nor external representations perceived by, for example, proprioception. Both of these representations can be directly perceived, selected, and maintained as internal depictive or embodied representations.

An immediate consequence is that depictive and embodied representations may directly contribute to mental model construction (through parsing or structure-mapping), whereas descriptive representations contribute to mental model construction only after their symbol structures are decoded and their meaning has been extracted. This is because both types of internal representations (depictive and embodied) are conceived as analogue representations of the mental model itself (e.g., Glenberg, 1997; Johnson-Laird, 1983). In contrast, symbol-based descriptive representations cannot be integrated directly into a mental model: Here, a surface representation or a verbal description needs to be translated into a list of propositions before these propositions can be used to specify a mental model (e.g., Van Dijk and Kintsch, 1983).

Both depictive and embodied representations are assumed to interact with propositional representations (e.g., Schnotz, 2022). Following ITPC, aspects of a representation can be translated into an explicit proposition. Thus, depictive representations may augment or modify the descriptive propositional representation. In the other direction, the descriptive propositional representation may help to interpret what is depicted in a picture and, thus, substantiate, focus, correct, or modify the internal depictive representation. Also, following ideas of embodied cognition (Barsalou, 2008, 2020), symbolic processing may not be independent of an internal sensorimotor system that is related to embodied representations. Here, conceptual and propositional processing is implemented via modality-specific simulations, such that embodied representations can give rise to symbolic structures, and, conversely, symbolic processing contributes to embodied representations.

Also relevant for the working memory system, mental model construction receives input from (a) depictive representations, (b) embodied representations, (c) descriptive propositional representations, and (d) long-term memory. Construction processes can be triggered by each type of representation (a, b, and c) and prior knowledge (d). As explained in detail by Erlebach and Frank (2022), prior knowledge and skills also influence corresponding processing in working memory (as well as the extended perception processes of sensory memory). In cases (a) and (b), the structure of an internal depictive and/or embodied representation is mapped on a mental model that is no longer bound to one specific source. Particularly in the fields of language learning (Dijkstra and Peeters, 2023) and acquiring mathematical concepts (Khatin-Zadeh et al., 2022), empirical evidence also indicates that embodied representations are important for developing mental models. In cases of descriptive propositional representations, a set of propositions is translated into a mental model that represents a typical situation compatible with these propositions. In this process, long-term stored knowledge needs to be integrated, as propositions often imply information that needs to be activated from long-term memory. If such (prior) knowledge is unavailable, propositions cannot be translated into a mental model and will likely be discarded or related to other existing mental models. Here, it again becomes clear that even knowledge-driven, top-down processes strongly influence multimedia learning (Erlebach and Frank, 2022). Alternatively, comparable constraints can be induced from an internal depictive or embodied representation. Therefore, lacking prior knowledge can (partially) be compensated for by additional external representations. Thus, with an increasing number of related representations, the basis for constructing a coherent mental model grows.

The key challenge in this process is determining whether the emerging mental model is compatible with all external representations. The more different external representations are available, the more opportunities one gets to check the mental model’s validity. With each step in the construction of the mental model, one can inspect and check whether the mental model’s current state still corresponds with the propositional representation. Inconsistencies may result in a modified mental model in which elements of the mental model are revised with respect to specific propositions. If an internally consistent model (i.e., if the model is consistent with all external representations) conflicts with prior knowledge, then long-term knowledge may be modified—a process we usually call “learning.” Here, the establishment of coherence, as described by Seufert (2003) and mentioned in section 2.2, once again takes on great significance.

Finally, we consider, according to Halford et al. (1998), that the working memory system has a limited capacity, and the CTML and ITPC also assume a limited capacity per individual channel/branch. Such capacities are conceptualized as “the number of independent dimensions that can be related in parallel” (relational complexity) rather than by the amount of information, such as the number of items that can be held in working memory simultaneously (Halford et al., 1998, p. 829). Referring to Baddeley, Baddeley’s (1986, 2000) model, relational complexity taxes the central executive components rather than the storage components of working memory. This idea was supported by studies validating working memory measures in which researchers focused on coordinating information elements into more coherent structures (e.g., Oberauer et al., 2000; Oberauer et al., 2003). In studies using a single external representation (sentences or text), relational complexity of the input was shown to tax working memory capacity. These studies demonstrated that in text comprehension, (Dutke et al., 2010, Dutke and von Hecker, 2011), deductive reasoning (e.g., Oberauer et al., 2006), and figural transformations (e.g., Mayr and Kliegl, 1993), high coordinative working memory capacity helped participants efficiently constrain the emerging mental model.

Another implication can be found for the model integration process, where two differently accentuated sub-processes can be distinguished. As a kind of default orientation, learners first aspire to a global understanding (McCrudden and Schraw, 2007), such that learners may first obtain semantic coherence among the different input representations and the emerging mental model. Upon obtaining a global understanding of what the learning materials are referring to, more specific learning goals may emerge, or more specific learning tasks may come into focus. This implies that the initially constructed mental model is adapted to these goals and task demands (Zhao et al., 2020). Next, adaptive mental model specification requires further interaction between symbolic, iconic, and embodied working memory representations by increasingly constraining the mental model (Dutke et al., 2010). Effectively constraining the mental model is particularly important, as multiple working memory representations increase cognitive load. Therefore, rapidly discarding possible but, in this situation, irrelevant variants of the mental model is essential to reduce unnecessary cognitive load.

In these two sub-processes, text and pictures play different roles, as described by Zhao et al. (2020). In initial mental model construction, processing text-related information dominated picture-related information, whereas during adaptive mental model specification, more resources were spent on processing picture-related information compared to text-related information. The authors argued that during initial model formation, the text creates inter-representational coherence more efficiently than a picture because text presents specific propositions in a particular sequence, which provides more constraints than mapping an internal mental picture on the mental model. For adaptive model specification, however, a picture is more suitable because it provides non-sequential access to those components relevant to the specific adaptation to be performed.

However, Cohn (2016) demonstrated that pictures and other iconic external representations can be reconstructed as “grammatically” structured sequences. The complexity of these grammatical structures is crucial for the question of how external representations from different modalities interact. Against this background, the finding by Zhao et al. (2020) that text information dominated the picture information during initial model construction might represent only one of many possible types of multimodal interactions.

These statements regarding text and pictures can be extended to other external representations that broaden the range of involved sensory modalities. In this context, the general dominance of text is partly called into question when additional modalities are considered. For example, studies provide evidence for the dominance of gestures (Hsu et al., 2021), which are sometimes interpreted as priming language processing (Zhao, 2023). Overall, these findings suggest that expanding the range of sensory modalities also calls into question the dominance of any single modality in the construction and evaluation of mental models. At the same time, it should be noted that the current empirical evidence does not yet provide conclusive results.

#### 4.1.4 Practical example as an illustrative application of the model extension

This section explains different pathways of learning from external representations. These pathways exemplify how the current theories on learning with multiple representations can be extended so that they apply to different combinations of external representations. We present two scenarios as examples for how students could be taught the *law of lever*.

In the first scenario, we assume that the *law of lever*, a fundamental principle of mechanics, is learned from a graphical illustration visually describing the relation of mass, lever arm, and acting forces by including force arrows in an illustrative representation of a lever. Figure 4 illustrates related pathways of cognitive processing. Initially, the graphical illustration is perceived through sight (marker 1). Information is selected and transformed into a depictive representation in working memory (marker 2). Even though this internal representation potentially contributes to mental model construction in a direct manner (marker 3.1), it may be that (parts of) the depictive representation is transformed into a (propositional) descriptive representation (marker 3.2). This, in turn, can also support the construction of the mental model directly (marker 4.1) or enables transformation into an internal embodied representation that could be directly reconstructed into movement patterns associated with proprioceptive stimuli (marker 4.2). Depending on the respective cognitive process, the internal embodied representation can be integrated into an (existing) mental model or will be attended to specify or inspect a mental model (marker 5).

**FIGURE 4:**
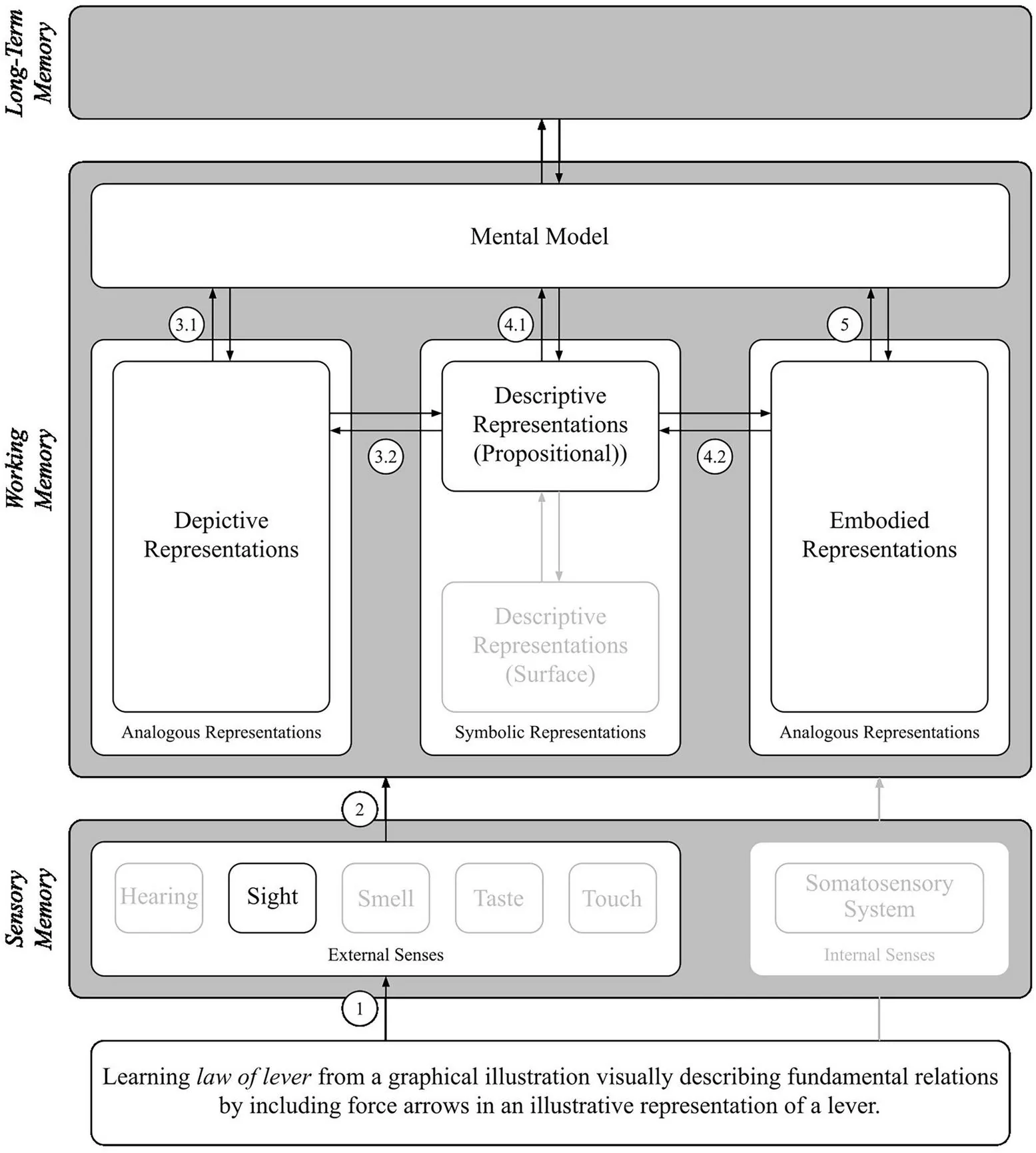
Exemplary application of the theoretical extension: Learning a fundamental principle of physics (law of lever) from a graphical illustration.

In the second scenario, the *law of lever* is learned during a class discussion in which the fundamental relations are discussed verbally and the acting forces can be physically experienced by the learners in experiments at the same time. Figure 5 illustrates related pathways of cognitive processing. Initial instructions are perceived through hearing (marker 1.1) or proprioception via feeling the acting forces physically (marker 1.2). Next, information becomes selected and transformed into a descriptive and an embodied internal representation (markers 2.1, 2.2). Processing of the internal depictive representation includes analyzing the surface characteristics and subsequently examining propositions (marker 3). Due to the asymmetric processing for symbolic and non-symbolic representations in working memory, this step does not occur when analyzing the internal embodied representation. Instead of transforming the (propositional) descriptive representation into an embodied representation (or vice versa), one compares the internal descriptive and embodied representations, primarily to detect inconsistencies (marker 4). Finally, both internal representations contribute to mental model construction (markers 5.1, 5.2).

**FIGURE 5:**
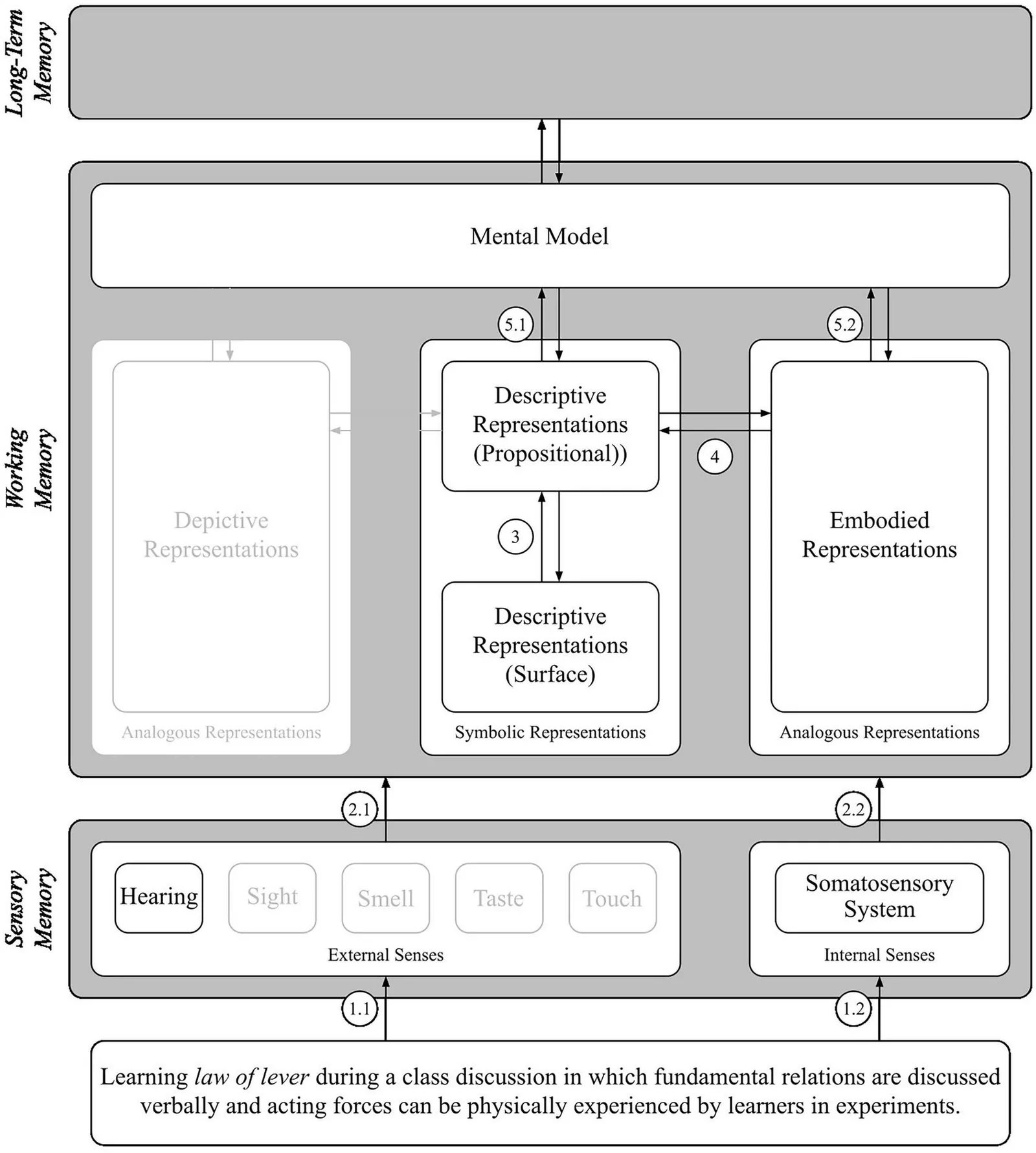
Exemplary application of the theoretical extension: Learning a fundamental principle of physics (law of lever) from a verbal discussion and the simultaneous physical execution of experiments by learners.

### 4.2 Implications: rethinking principles of multimedia learning

So far, we have discussed extending the existing theoretical approaches with respect to their theoretical foundations in cognitive psychology. In turn, though, this theoretical extension is meant to provide implications for further empirical studies on multimodal learning.

The subsequent sections refer to three widely known principles of multimedia learning (modality principle, spatial contiguity principle, and temporal contiguity principle) related to CTML (Mayer, 1997, 2022) and discuss potential questions and ideas for research on extended principles of multimedia learning derived from the theoretical extension presented above. These considerations are not exhaustive but represent exemplary ideas. While additional principles of multimedia learning would also likely benefit from similar in-depth analysis, examining all the diverse principles within this context is outside the scope of the present contribution. Therefore, the examples we give below provide potential questions to encourage further research and to inspire researchers to propose and investigate new hypotheses related to this multimodal framework for learning. Further, as the ITPC offers very specific descriptions of processes, this should allow researchers to derive and empirically test certain boundary conditions under which the principles are valid.

#### 4.2.1 The modality principle(s)

Considering the background of the modality principle(s) already discussed in section 2.1. and following the theoretical extension, hypotheses for extended principles of multimedia learning with respect to the modality principle(s) can be derived. First, the limited capacity and highly limited time that information is stored in the sensory memory systems mean that perceiving external information through different sensory modalities will increase the probability that information is selected and processed. Consequently, learning can be supported by providing learners with information through different external and internal senses.

Second, embodied representations may foster learning if interactions between embodied representations and descriptive or depictive representations increase the germane processing load and do not increase extraneous cognitive load. Different investigations have demonstrated that even (typically visual) perceptions of full-body activities or gestures without motoric or proprioceptive stimuli can foster learning. This applies to observing human movements or gestures, such as tying a knot (Marcus et al., 2013), and it also applies to visually perceiving non-human movements or dynamic processes (e.g., De Koning and Tabbers, 2013; Brucker et al., 2015). Appropriately in this context, Goldin-Meadow and Alibali (2013, p. 261) discussed gestures as actions that open up “a second channel that makes successful comprehension more likely.”

Therefore, it seems reasonable for further studies to investigate whether people learn better via stimuli from internal senses and the somatosensory system, for example, motoric and proprioceptive information, in combination with stimuli from external senses, for example, pictures or (written or spoken) words, or via stimuli from external senses alone.

However, this very general formulation must always be understood within the context of possible boundary conditions; the extension to other sensory modalities constitutes the central research concern in connection with the modality principle, see section 2.1.

Another potential conclusion from the theoretical extension related to modality effects refers to the outlined dominance of sensory impressions (Zhao et al., 2020). Depending on whether a mental model is initially constructed or adaptively specified, constraining external representations could foster learning. Whereas stimuli from external senses are directly constrained due to their presentation, stimuli from internal senses and the somatosensory system cannot be narrowed to the same extent because motoric and proprioceptive information can only be influenced to a certain degree. Therefore, stimuli from internal senses could help students to examine and specify mental models effectively. Empirical studies, for example, within physics education research (Laumann and Heusler, 2018), have demonstrated that students find it easier to correct existing, long-held concepts if they additionally perceive appropriate stimuli from internal senses that counter their former concepts. Furthermore, stimuli from internal senses in the form of motions and motoric signals are processed as embodied representations within working memory and are potentially transformed into descriptive or depictive representations. Thus, the probability of detecting inconsistencies in mental models may increase with the number of external representations. Restricting these external representations to words and pictures could impede extensive processes of model inspection. However, as the current empirical evidence does not yet provide conclusive results, future studies may investigate whether people adapt existing concepts more easily based on new stimuli from internal senses and the somatosensory system, for example, motoric and proprioceptive information, or both via stimuli from internal and external senses, such as pictures or (written or spoken) words, or via stimuli from external senses alone.

#### 4.2.2 The spatial and temporal contiguity principles

To extend the spatial and temporal contiguity principles, two distinctions are helpful: First, related to the temporal dimension, principles of multimedia learning can either indicate simultaneous or sequential presentations. Second, related to the temporal and spatial dimension, the content being considered in different types of representations can be either equivalent or complementary. According to the traditional contiguity principles and also more generally in line with the theoretical extension, spatial contiguity often fosters learning and helps students develop mental models efficiently. Consequently, learning outcomes are positively affected by immersive learning environments that embed perceptions of different external senses or external and internal senses in direct spatial proximity. This effect can be explained by the coherent presentation, which helps learners integrate specific information from the individual representations into a mutual mental model if germane cognitive load is increased. This resembles the traditional spatial contiguity principle, “learners do not have to use cognitive resources to visually search page or screen” (Mayer, 2009, p. 135) and generalizes it by supposing that spatial contiguity should also be considered for non-auditory and non-visual representations to achieve greater learning gains. Also, the quality of learners’ mental models may increase with a greater amount of input information, which could help learners detect inconsistencies in their mental models. Thus, studies could investigate the extent to which learning outcomes are influenced by whether corresponding stimuli are presented close to rather than far from each other.

Another conclusion of the theoretical extension is closely related to engaging sensory modalities in the temporal dimension. Although perception and processing take place within different channels depending on the involved sensory modalities (or types of representations), there is a danger of overloading the sensory system. Another challenge is that when numerous external representations are present, the process of mental model inspection requires increased cognitive load. Therefore, it might be important to present complementary content simultaneously, because transformations between different internal representations and continuous comparisons can foster bottom-up construction of a consistent mental model. By contrast, sequentially presenting content could cause learners to construct less compatible mental models, further taxing their working memory due to representations processed from different stimuli of external senses. Here, additional cognitive load could result from inspecting the mental model and uncovering inconsistencies. It may be of interest for future research to investigate whether people learn better through a specific number of (external and/or internal) sensory modalities when complementary content is presented and perceived simultaneously compared to learning where complementary content is presented and perceived sequentially. Given that constraints strongly affect the principles of multimedia learning, as has been noted on several occasions, it seems worthwhile to analyze whether too few modalities might make it difficult to integrate information, while too many modalities might cause cognitive overload.

## 5 Discussion

Multimodal learning is highly important for traditional instructional settings and more recent approaches are integrating a wide range of modern educational technologies that rely on multiple representations. The sensory modalities of hearing and seeing appear to be particularly important for learning through multiple representations, which is why theoretical frameworks describe multimodal learning processes accordingly. In particular, the cognitive theory of multimedia learning (CTML) and the integrated model of text and picture comprehension (ITPC) describe such processes and practical conclusions. CTML provides a well-founded account of limited processing capacities and cognitive load in multimedia environments, while ITPC focuses on the integration of different representational formats. However, the theoretical focus of both frameworks, as well as the emphasis of related empirical studies, lies on bimodal processes, primarily addressing seeing and hearing. Despite the numerous advantages of this focus, it does, to a certain extent, limit the range of learning environments for which CTML and IPTC provide explicit guidance.

As such, we noted that many learning environments also involve haptic interactions and whole-body engagement as part of the learning process and, therefore, cannot be fully captured within a strictly visual-auditory framework. Therefore, the extension proposed in this paper examined how existing approaches (CTML, ITPC) could be expanded by taking into account a broader spectrum of modality-specific perceptions at the level of sensory memory and by discussing the interplay between descriptive, depictive, and embodied representations in working and long-term memory.

It is important to note that this proposed extension of existing theories should not be understood as the introduction of a validated new model. Rather, it is a theory-driven extension that combines assumptions from CTML and ITPC with findings from research on, for example, embodied cognition and further insights into embodied representations. Its primary goal is to provide a conceptual framework that helps formulate hypotheses and structure future empirical work. Furthermore, the conceptual framework should be interpreted considering its exploratory and heuristic nature. Against this background, the theoretical contribution of this paper lies less in replacing existing conceptual frameworks and more in expanding their scope of application. The present approach combines the specific perspectives of CTML and ITPC and extends them to include additional sensory modalities and embodied forms of representation.

The research questions posed here explore how existing theories can be expanded and what implications such an expansion might have for empirical studies and, ultimately, for the design of learning environments. The analysis suggests that an expansion is plausible under certain conditions, particularly when additional sensory modalities provide complementary, task-specific information that can be integrated into a coherent mental model. Building on this theoretical expansion, further research questions arise for future studies, for example, regarding the learning benefits of simultaneously addressing different sensory modalities or the influence of available cognitive resources on the learning-related impact of presenting additional information.

However, the issues raised also highlight an important issue of the proposed model extension: Additional sensory modalities can support learning if they are appropriately aligned with the learning objective and if they contribute unique information to the representational system. Conversely, they could increase cognitive load or impair model construction if the relevance of additional representations is unclear or coordination between sensory modalities is challenging. For beginners in particular, multimodal enrichment can quickly exceed available cognitive capacities, while more advanced learners can benefit from the same features. The discussion of the existing literature within this contribution emphatically illustrates that considering boundary conditions is of enormous importance and makes general statements difficult. The impact of cognitive load on learners—whether it is too low or too high—and the ambivalence surrounding hypotheses about multimodal learning should therefore be taken seriously: The proposed extension does not resolve the tension between the richness and efficiency of representation but rather makes it more explicit.

A limitation of this contribution concerns the interpretation of empirical effects related to multimodal and embodied learning environments. The observed benefits of movement, gestures, or haptic interaction may not be attributable exclusively to embodied representations. Furthermore, follow-up studies need to be conducted more extensively in conjunction with a theoretical extension to gather structured evidence regarding the validity of the theoretical extension. Without more systematic evidence, it remains only hypothetical that embodied representations specifically contribute to the construction of mental models.

Although the present framework is primarily conceptual, it lends itself to concrete empirical investigations of precisely these questions and assumptions. For example, studies could compare traditional text-image conditions with conditions that additionally involve haptic or motor interaction, vary the temporal coordination of multimodal inputs, or manipulate the degree of motor relevance in a task. The discussed example of the law of levers illustrates how such experimental designs could be implemented in practice.

As such, the implications derived from this conceptual framework for future studies—particularly regarding the principles of multimedia learning—should be interpreted at most as hypotheses, and certainly not as equally expanded and established principles. While existing principles of multimedia learning offer a useful starting point, their extension to additional sensory modalities requires systematic validation. It seems plausible that principles such as coherence, signaling, or redundancy may need to be reformulated when applied to haptic, proprioceptive, or action-based representations. However, this paper can only outline these perspectives and does not yet offer empirically grounded guidelines.

In practice, the relevance of additional sensory modalities for learning processes will likely vary depending on the subject area. In fields such as science education or medicine, where spatial relationships and physical properties are central, haptic or motor information can provide valuable support for understanding. In other fields, however, such information may be superfluous or even distracting. Thus, practical recommendations must always be context sensitive and take into account the characteristics of individual fields. This also appears relevant for empirical studies, which should, therefore, also be conducted across various fields.

Finally, despite the relatively broad scope of this contribution and its incorporation of findings from various disciplines, it is important to identify key areas for future research. Three directions appear particularly crucial in this regard: First, it seems important to empirically validate the proposed assumptions regarding multimodal and embodied processing; second, to systematically investigate boundary conditions, for example, regarding cognitive load and prior knowledge; and third, to clarify the cognitive processes underlying the integration of multiple sensory modalities. Addressing these questions would significantly strengthen the theoretical foundations of the proposed extension.

Overall, this paper aims to broaden the perspective of research on multimodal learning. Its main value lies in formulating a series of theoretically grounded questions and identifying the conditions under which an expansion beyond visual and auditory sensory modalities can be fruitful. Whether and to what extent such an expansion can be empirically substantiated remains an open question and a central task for future research.

## Data Availability

The original contributions presented in the study are included in the article/supplementary material, further inquiries can be directed to the corresponding author.

## Footnotes

1 Due to differing terminology and concepts in the aforementioned theories, the following two sections on CTML and ITPC initially use the original terminology in each case. As we further develop the theories, the terms we subsequently use are explicitly introduced and explained at the beginning of section 4.

2 It should be noted that working to further develop theoretical foundations is compatible with ongoing developments in the field. For current research on affective-motivational variables, we refer readers to Schrader et al. (2022); for current research on prior knowledge-guided, top-down processes in multimedia learning, to Erlebach and Frank (2022).
